# The Impact of Weather Conditions on Biocides in Paints

**DOI:** 10.3390/ma15207368

**Published:** 2022-10-20

**Authors:** Ute Schoknecht, Helena Mathies

**Affiliations:** Federal Institute for Materials Research and Testing, Unter den Eichen 87, 12205 Berlin, Germany

**Keywords:** substance release, construction products, biocides, transformation, weathering, driving rain, global radiation

## Abstract

Weather conditions affect biocides on exposed outer surfaces on constructions. Contact with water causes hydrolysis and leaching of substances. Ultraviolet radiation may induce photolysis. As a result, a mixture of biocidal active substances and transformation products can be emitted into the environment. In a semi-field study, leaching of the biocidal active substances terbutryn, diuron, octylisothiazolinone, carbendazim, and selected transformation products was observed for two paints containing either a white or a red pigment. Painted test panels were exposed to natural weathering for about 1.5 years. Runoff samples were analyzed during the course of the experiment. At the end of the study, residues of biocidal active substances and transformation products were determined in sections of the test panels. Emissions of substances were mainly observed during the first few months of the experiments. Increased emissions of transformation products were observed during periods of increased global radiation and subsequent periods with relatively high amounts of driving rain. Different patterns of transformation products were observed, especially for terbutryn, both for paints containing different pigments and in experiments that were started in different periods of the year, as well as during different periods of the experiments.

## 1. Introduction

Biocidal active substances (hereafter referred to as active substances) are often added to coatings to protect them from algal and fungal growth. When exposed to weathering, these substances in the rather thin coating layers can be transformed by photolysis and hydrolysis, and the active substances, as well as transformation products, may leach into runoff water [1,2,3,4,5,6,7,8,9,10]. This means that not only the original active substances, but also transformation products, can contribute to the environmental impact of biocidal products. This is taken into consideration in the European Biocidal Products Regulation (BPR) [11], which requires environmental assessment of ecotoxic transformation products. However, such assessments require knowledge about possible transformation products and the conditions under which emissions of these substances into the environment can be expected.

In early studies on the emission of biocides from coatings [1], the dealkylation product of terbutryn “M1” was included. Later, the transformation of terbutryn and octylisothiazolinone (OIT) in coatings was investigated in detail [2,5]. Transformation products of diuron due to photolysis are known from investigations of the substance in aqueous solution [12,13]. Carbendazim is presumed to be relatively stable, although a hydrolysis product has also been described [14]. Several authors have reported the occurrence of transformation products of active substances that are applied in coatings in the environment [6,9,10]. The occurrence of active substances under varying weather conditions was investigated by event-based monitoring of a stormwater catchment for a period of nine months [15]. Emissions of terbutryn, OIT, and its transformation products were observed during a semi-field study on ETICS (external thermal insulation composite systems) for a duration of 20 months under natural weather conditions [2,5]. It was demonstrated that pigments have an effect on the photolysis of terbutryn, diuron, and OIT in paint films, whereas carbendazim is less affected by exposure to UV radiation under laboratory conditions [7]. In an extended laboratory experiment that included periods of water contact in combination with exposure to UV radiation, it was demonstrated that water contact and UV radiation mutually influence transformation processes and leaching of substances [8]. Transformation of terbutryn due to photolytic and hydrolytic reactions and biodegradation was investigated in laboratory studies [16]. Current knowledge regarding biocide emissions from building materials during wet weather has been summarized by Paijens et al. [17].

Earlier semi-field studies on the emission of active substances from paint films [3,4] showed that the progression of emissions differs for different active substances. A steep increase in emissions was observed for terbutryn, diuron, and OIT at the beginning of these experiments. During later intervals, the emissions of diuron decreased only slowly and were clearly visible for up to one year, while emissions of terbutryn and OIT declined substantially after the initial phase. This observation indicates that transformation of substances can impact leaching processes. In addition, the course of the emissions was not only dependent on the amount of runoff, but included periods of delayed and accelerated leaching, which continued when the experiments were extended to four years [18]. Repeated tests at different sites and time periods resulted in different emission behaviors for the individual experiments. Regression analysis showed that these different courses could be mathematically described based on actual weather data. Several weather parameters proved to be important, i.e., the amount of precipitation, wind direction, and velocity determining the amount of driving rain, as well as temperature, relative humidity, and global radiation [3]. The importance of global radiation values led to the conclusion that transformation processes affect the course of emissions.

This study investigated the influence of natural weather conditions, especially precipitation and global radiation, on transformation and leaching of active substances from film preservatives in paint films. The following questions were considered: (1) Which transformation products can be observed under outdoor conditions? (2) Do precipitation and global radiation influence emission courses of active substances and transformation products? (3) Do pigments in the paints influence the transformation of active substances? (4) Are observations in laboratory and semi-field experiments similar?

The investigated paint formulations were identical to those used in laboratory studies [7,8]. A white paint contained titanium dioxide and a red paint contained hematite. The paint formulations included the active substances terbutryn, diuron, OIT, and carbendazim. These substances belong to the active substances that are used in film preservatives. While carbendazim has already been approved under the BPR regulations, the evaluation of the other three substances is still ongoing (see [19] for information on biocides).

The paints were applied on birch plywood panels and exposed to natural weathering for about 16 to 18 months, starting at two different times—summer and beginning of autumn. Runoff samples were analyzed for the four active substances and selected transformation products to observe the release of substances. At the end of the exposure period, the same set of analytes was measured in sections of the test panels, including the paint layer and the uppermost part of the wooden panel. The results of this study demonstrate that transformation of active substances in coatings and emission into runoff are affected not only by the availability of water, but also by global radiation.

## 2. Materials and Methods

White and red styrene acrylate paints, containing either titanium dioxide (TiO_2_: white paint) or hematite (α-Fe_2_O_3_: red paint) were prepared and provided by the Dr. Robert-Murjahn-Institut GmbH (RMI), Ober-Ramstadt, Germany. The paint formulation was developed for leaching tests with systems of organic renders and paints. It has been verified that it can be applied to other substrates as well. The paints contained various active substances that are commonly used in film preservatives, i.e., diuron, terbutryn, octylisothiazolinone (OIT), and carbendazim at final concentrations of 500 mg kg^−1^. The active substances were not encapsulated (see Supplementary Information SI S1 for the composition of the paints).

Test panels of birch plywood of about 80 cm × 80 cm were used for the semi-field experiments. The edges of the boards were sealed using ‘Pyrotect Lack’. An acrylic dispersion that did not contain film preservatives, but other active substances as in-can preservatives, was applied manually as a primer (150 g m^−2^ to 160 g m^−2^). Afterwards, two layers of the paints were applied manually on two consecutive days in accordance with the manufacturer’s instructions (440 g m^−2^ to 460 g m^−2^) (see Supplementary Information SI S2 for details on the test panels). Two layers of biocide-free red paint were applied on the red test panel for experiment A (460 g m^−2^), followed by two layers of red paint that contained biocides. The paints were applied with rollers. The applied amount was controlled by weighing the paint. The test panels were kept inside under ambient conditions (22 °C +/− 2 °C and 50% to 60% relative humidity) for 6 to 14 days prior to the start of the outdoor experiment.

The test panels were installed vertically oriented on the wall of a low building, facing south-southwest (225°), i.e., driving rain arriving within the range of (225 − 90)° to (225 + 90)° could impinge on the test surfaces at sufficiently high wind velocities. The runoff water was collected in stainless steel channels and directed into glass bottles. The runoff in the glass bottles was sampled and analyzed for active substances and selected transformation products after each rain period (maximum duration three days due to weekends). Samples that could not be analyzed directly, were stored at −18 °C until analysis.

Weather data at intervals of 10 min, i.e., rain amount, temperature, wind direction and velocity, relative humidity, and global radiation, were obtained from a weather station operated by the DWD (Deutscher Wetterdienst, Potsdam, Germany) and located in the vicinity of the test panels.

After completion of the semi-field exposure, the residual amounts of the active substances and selected transformation products were determined in the paint film and in the uppermost layers of the plywood matrix. Sections measuring 2 cm × 2 cm were cut from six positions of each panel: two sections from the upper part, two sections from the middle and two sections from the lower part.

Layers of 20 µm were removed from the paint film and the plywood matrix by a microtome (MICROM HM440E, Microm Laborgeräte GmbH, Walldorf, Germany). All cuttings from the paint film were combined into one sample. The plywood cuttings from each 1 mm thick layer of the matrix were combined to give three samples (0 to 1 mm, 1 to 2 mm, 2 to 3 mm). The samples were extracted with methanol (0.22 ± 0.07 g cuttings from the paint film (mean ± standard deviation, *n* = 24) in 15 mL methanol and 0.34 g ± to 0.08 g cuttings from wood (mean ± standard deviation, *n* = 72) in 10 mL methanol) via sonication for one hour at 40 °C and 100% performance (SonorexSuper 10P, Bandelin, Berlin, Germany). After filtration through syringe filter holders (cellulose, 0.2 µm), the extracts were analyzed for the active substances and transformation products via liquid chromatography combined with mass spectrometry (LC-MS).

In addition to the four active substances, five transformation products of terbutryn, two transformation products of diuron, and three transformation products of OIT were quantified in the runoff samples and extracts from the test specimens (see Table 1 and Supplementary Information SI S4). The diuron transformation products, monuron and dichloroaniline, were determined in the extracts from the test specimens and in a few runoff samples at the end of the experiment. The inclusion of transformation products into the test program was motivated by literature data [2,5,7] and our own studies [8]. See Supplementary Information SI S3 for details on the LC-MS analysis of runoff samples and extracts from test specimens. Structures, molecular formulas, and masses of the analyzed substances are listed in Supplementary Information SI S4.

The emission per unit area *E_i_* was calculated for each runoff sample using the measured concentrations in the runoff samples *c_i_*, the volume of the runoff samples *V_i_*, and the area of the test panels *A* according to Equation (1). Cumulative emissions *E*_cum_ were calculated as the sum of *E*_1_ to *E_i_*.

(1) $$E_{i}\;\left[mg\;m^{-2}\right]=\frac{c_{i}\;\left[mgl^{-1}\right]\times V_{i}\;\left[l\right]}{A\;\left[m^{2}\right]}$$

The residual amount per unit area *R* was calculated using the concentration in the extract, the extract volume, and the area of the test section according to Equation (2). Mean values were calculated for *n* = 6 test sections. The sum of residues in the paint film and plywood layers up to 3 mm was used to calculate mass balances.

(2) $$R\;\left[mg\;m^{-2}\right]=\frac{c\;\left[mgl^{-1}\right]\times V\;\left[l\right]}{A\;\left[m^{2}\right]}$$

## 3. Results and Discussion

### 3.1. Weather Conditions during the Outdoor Experiments

The semi-field test was performed in the southwestern part of Berlin with a temperate climate and yearly precipitation of 591 mm (mean of 1981 to 2010 for Berlin-Dahlem) [20]. The test panels of experiment A were exposed to an unusually high amount of heavy rain (about 160 L m^−2^) during the second week of the experiment in July 2017. In addition, in August and in the autumn of 2017, the amount of rain was relatively high (94 L m^−2^) compared to mean values for Berlin-Dahlem (see Supplementary Information SI S5). In contrast to 2017, rainfall was below, and global radiation was above, the long-term average during the summer and autumn of 2018. The calculated monthly driving rain ranged between 3% and 34% of the monthly precipitation. The high initial amount of rain during experiment A was the reason to start a second experiment (experiment B) in September 2017 and take the opportunity to investigate leaching and transformation of substances under different weather conditions. Table 2 summarizes precipitation, global radiation, and runoff from the test panels during the experiments.

The weather conditions during the semi-field study can be roughly divided into the phases described in Table 3. Low precipitation and high global radiation in summer, like in 2018, were also observed at the site in subsequent years. Test panels of experiment B were not exposed to Phase 1.

Admittedly, high amounts of global radiation are usually correlated with higher temperatures. That means that temperature-dependent chemical reactions and transport processes, as well as evaporation, are increased in addition to photolysis caused by the photochemically active spectrum of the global radiation. This effect was not considered in detail during this study.

For a few days, surface temperature was measured on the panels and compared to the air temperature. In the morning, the temperature of the test panels was slightly below the air temperature. During the day, the temperature on the panels increased to values above the air temperature, with higher temperatures on the red panel (up to about 70 °C) than on the white panel (up to about 50 °C) (also see Supplementary Information SI S6).

### 3.2. Concentration of Active Substances and Transformation Products in Runoff Samples

The investigated substances were all detected in runoff samples, with the exception of the terbutryn transformation product terbumeton. Terbutryn itself was not detected in 16 white panels and 17 red panels of the 83 samples in experiment A, and in 8 white panels and 10 red panels of the 69 samples of experiment B, although transformation products were present. TB-DesE, TBOH-DesE, DCPU, OIT, OAM, OOA, and OMA were not detected in some of the runoff samples.

The concentrations of terbutryn, TB-DesE, TBSO, and diuron exceeded German environmental quality standards in a large number of samples, indeed in all samples for diuron (see Supplementary Information SI S7, Table S1). That means that runoff from these test panels needs a high dilution factor to meet the environmental quality standards. Due to the geometry and exposure situation of the test panels, the measured concentrations and required dilution factors are not representative of real buildings. The test conditions were designed to represent a worst-case situation.

A tendency for concentrations to decrease with duration of the semi-field experiment was observed for the active substances terbutryn, diuron, carbendazim, and OIT, and for the OIT transformation products OAM, OOA, and OMA. For some of the terbutryn transformation products, i.e., TB-DesE (experiment A), TBOH, and TBOH-DesE, clear increases in the concentrations in runoff samples were observed during the summer and the autumn of 2018. The concentrations of TB-DesE in the runoff samples decreased during experiment B. The concentrations of TBSO increased in the runoff samples from experiment B during April and May 2018. The concentrations of the diuron transformation product DCPU in runoff samples of both panels and of DCPMU in runoff samples of the red panel remained in a fairly constant range during both experiments. The DCPMU concentrations in the runoff samples of the white panel increased during the summer and the autumn of 2018 in experiment A and during autumn 2018 in experiment B. Another increase of concentrations of transformation products, especially of TBSO, TBOH, TBOH-DesE, and DCPMU, was observed for runoff samples in December 2018. Dichloroaniline and monuron were only measured for the last twelve runoff samples collected during December 2018 and January 2019. Both substances were detected only in a few samples at very low concentrations. Therefore, trends could not be derived for these substances. The measured concentration ranges are presented in Supplementary Information SI S7.

In many cases, the concentrations in the runoff samples were similar in the white and the red panels. Exceptions were carbendazim (experiment A); TB-DesE (experiment A); TBOH and TBOH-DesE (both experiments), with higher concentrations in the runoff samples from the red panels compared to the white panels; and DCPMU, with higher concentrations in the runoff samples from the white panels compared to the red panels.

However, the concentrations in the runoff samples were influenced by the actual volume of the runoff samples, which ranged between 5 mL and 3 L depending on the weather conditions. This difference can be relativized if the emissions per unit area *E_i_* are calculated and related to the amount of runoff.

### 3.3. Course of Emissions for Active Substances and Transformation Products

For the purpose of this study, the cumulative emissions *E_cum_* were not related to a parameter that describes the amount of water, like precipitation, driving rain, or the runoff volume, but to the dates. This made it possible to observe whether factors other than the availability of water influenced the course of emissions. To analyze this, the results need to be discussed in relation to the actual weather conditions (see Supplementary Information SI S5). Therefore, the emission data are presented in monthly intervals together with the cumulative runoff volumes and cumulative global radiation to facilitate the relation of observed emissions to actual weather conditions. The observed emission courses are shown in Supplementary Information SI S8.

**Figure 1:**
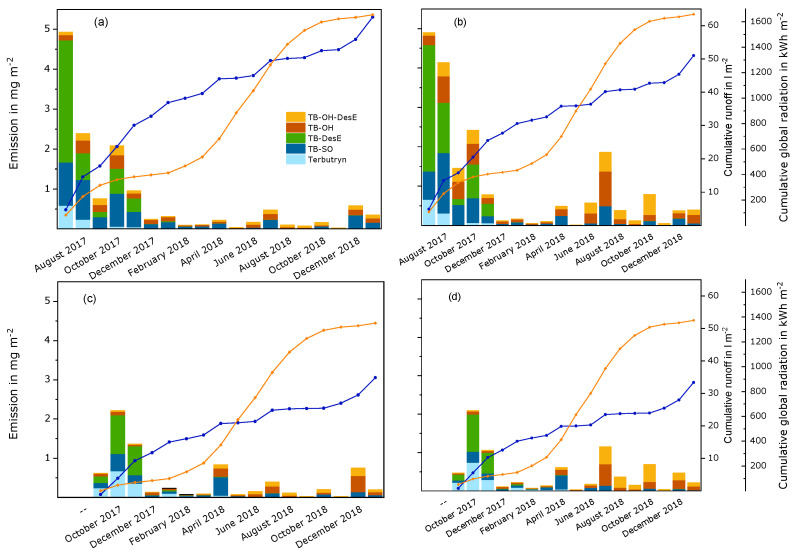
Monthly emissions of terbutryn and transformation products from a white and a red paint film during semi-field experiments that were started in July 2017 (experiment A) and September 2017 (experiment B). The columns represent the substances that were detected in runoff samples per month. The blue curves show the cumulated volume of runoff samples per surface area for each experiment. The orange curves show the cumulative global radiation during the experiment. (**a**) Experiment A, white paint; (**b**) experiment A, red paint; (**c**) experiment B, white paint; (**d**) experiment B, red paint.

Terbutryn and transformation products. During the semi-field study, about 6 mg m^−2^ to 20 mg m^−2^ of terbutryn were detected in runoff samples, either as the original substance or transformed. This amounted to about 3% to 8% of the initial quantity of the active substance in the test panels. It is remarkable that the amount of terbutryn in runoff samples was low compared to the transformation products, as can be seen in Figure 1 and Supplementary Information SI S8. Differences between both experiments and the two paints were observed. In experiment A, high amounts of rain caused high overall emissions during July and August 2017. The overall emissions decreased during autumn 2017, remained low during the winter 2017/2018—although the collected amounts of runoff were only slightly decreased—and increased again from early summer 2018. This increase was especially noticeable for the red panels compared to the white panels and occurred simultaneously with a steep increase in global radiation. Later, the overall emissions decreased, but appeared to depend on the runoff volume. For example, relatively high emissions in December 2018 and January 2019 correlated with high runoff volumes. In experiment B, the initial emissions were considerably lower than in experiment A. This correlated both with lower runoff volumes and lower global radiation at the beginning of the experiment. Later, the observations were similar to experiment A.

The emission curves of the different substances do not run in parallel. Terbutryn was mainly leached during the first two or three months of the experiments. Then, the emission curves flatten to almost no emission. TB-DesE and TBSO were the main transformation products at the beginning of the study. For TB-DesE, the emission curves flatten after December 2017 in both experiments. During the later phases of the experiments, especially from summer 2018, TBOH and the secondary transformation product TBOH-DesE dominated. TBSO was detected in all samples. The TBSO emission curves flatten after the initial phase but increase during certain periods of the study, i.e., in April, July, and December 2018. During these periods, relatively large volumes of runoff were collected. During April and July, there was also considerable input of energy due to global radiation. The emission curves of TBOH and TBOH-DesE show periods of steeper increase beginning in June and July 2018—when global radiation was especially high—and in October 2018, during a period with increased amounts of driving rain. This was mainly observed in both experiments on the red paint. The formation of TBSO, TBOH, and TBOH-DesE was probably accelerated due to high amounts of global radiation in spring and summer. The higher effect on the red paint film can be explained by the different absorption spectrum of the red paint film compared to the white paint film [7]. The increase of emissions in autumn and winter was probably caused by more intense water contact. Higher relative humidity delays drying, i.e., higher amounts of water are available in the paint film for transport processes, and higher driving rain induces transfer into runoff. A similar course of the leaching process was observed under laboratory conditions, where test specimens were exposed to the combined influence of UV radiation and water contact [8], i.e., the amount of terbutryn in the eluates decreased rapidly, and TBSO and TB-DesE were the main transformation products at the beginning of the experiment. During later phases, higher amounts of TBOH and TBOH-DesE were observed. Transformation occurred faster in the red paint film compared to the white paint film. Emissions of TBSO were similar for the white and the red panels in the semi-field study, whereas slightly higher amounts of TBSO were detected in the white paint film in the laboratory experiment.

Leaching of the terbutryn transformation products TB-DesE, TBSO, TBOH, and TBOH-DesE from an acrylate render and a silicone render were observed in Denmark during a similar period of the year, i.e., from August of one year until March of the next year [2]. In contrast to the results for the paint films, the amount of leached terbutryn was higher than the amount of the leached transformation products. This was probably caused by material properties. Due to their porous structure, renders are more absorbent for water than paint layers. In addition, ultraviolet radiation penetrates a smaller fraction of a render than of the thinner paint layer. As a result, terbutryn can be leached from deeper layers of the renders before it is transformed by photolysis. As in the study on paints, TB-DesE and TBSO were the main transformation products at the beginning of the experiment, whereas higher amounts of TBOH and TBOH-DesE were observed only later. After about one year—during summer and autumn—accelerated leaching was observed for all transformation products.

**Figure 2:**
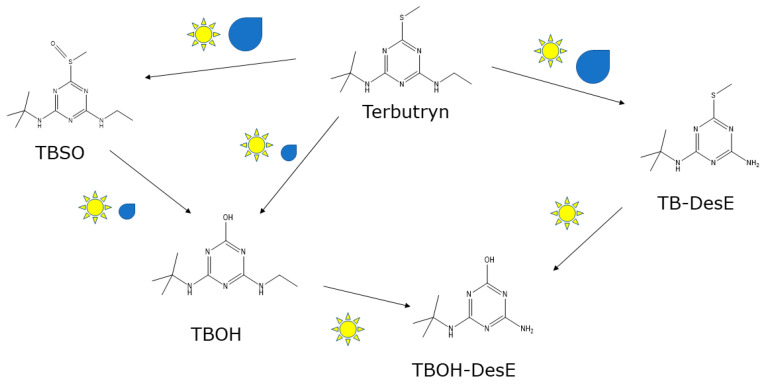
Transformation of terbutryn under natural weather conditions. The scheme includes only transformation products that were detected in this study. It indicates whether an increase of transformation products was observed after relatively large amounts of driving rain (symbol: water droplet) or global radiation (symbol: sun).

Observations on the influence of weather conditions on the occurrence of terbutryn transformation products in runoff are summarized in a simplified scheme (Figure 2).

A study comparing photodegradation, abiotic hydrolysis, and biodegradation of terbutryn under laboratory conditions used compound-specific isotope analysis (CSIA) to draw conclusions about reaction pathways [16]. In photodegradation experiments, the concentrations of TBSO and TB-DesE first increased and later decreased, while TBOH concentrations increased and reached a plateau. From the CSIA, the authors concluded that TBOH is preferably formed from TBSO, and TBOH-DesE is preferably formed from TB-DesE due to photolysis. Conclusions regarding a preferred pathway under natural weathering conditions are not possible based on the results presented here. Formation of TBOH-DesE was only supported by the UV radiation in the laboratory experiments [16]. This is in agreement with the observations for natural weathering conditions (this study) and a laboratory study on the transformation of terbutryn in paint films by combined influence of water contact and UV radiation [8]. Under laboratory conditions, TBOH was also formed by hydrolysis at very low and very high pH values [16]. This may be relevant for water-filled pores of alkaline renders rather than for the paint layers investigated in this study.

**Figure 3:**
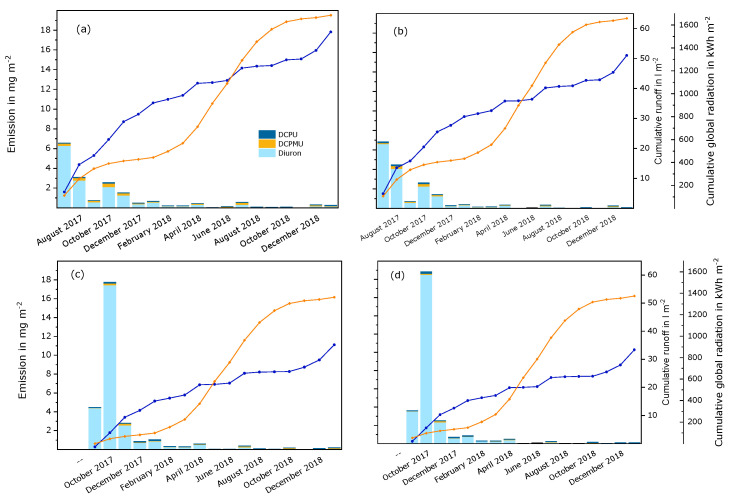
Monthly emissions of diuron and transformation products from a white and a red paint film during semi-field experiments that were started in July 2017 (experiment A) and September 2017 (experiment B). The columns represent the substances that were detected in runoff samples per month. The blue curves show the cumulated volume of runoff samples per surface area for each experiment. The orange curves show the cumulative global radiation during the experiment. (**a**) Experiment A, white paint; (**b**) experiment A, red paint; (**c**) experiment B, white paint; (**d**) experiment B, red paint.

Diuron and transformation products. About 18 mg m^−2^ diuron (around 8% of the initial amount) and transformation products were leached from both panels during experiment A (see Figure 3 and Supplementary Information SI S8). The emissions were relatively high at the beginning and decreased during the study. During experiment B, a considerably higher amount of diuron and transformation products, i.e., about 28 mg m^−2^ to 29 mg m^−2^ (about 13% of the initial amount), was leached. This was due to a large amount of diuron that was leached in October 2018 at the beginning of this experiment. After that, the emissions decreased to a level similar to experiment A. Diuron was the dominant substance in the runoff samples compared to transformation products. The emissions of the transformation products DCPMU and DCPU rose quickly at the beginning of both experiments, but to a higher extent in experiment A. This difference may be due to the differing amounts of global radiation at the beginning of the experiments. Another moderate increase was observed during summer 2018, when the panels were exposed to a high level of global radiation. In experiment A, the amounts of the primary transformation product DCPMU were slightly larger than the amount of the secondary transformation product DCPU, especially in the runoff from the white panel. In contrast to that, the emissions of DCPMU and DCPU were in similar ranges in experiment B.

It is possible that diuron was transformed in the period of high precipitation and moderate global radiation during summer 2017 into substances that were not investigated in this study, whereas it was more stable under the weather conditions at the beginning of experiment B. Analysis of the last twelve runoff samples of the experiments indicates that at least monuron and dichloroaniline were also leached from the test panels. Previous laboratory experiments on the same paints [8] have also indicated that transformation pathways other than demethylation can contribute to the transformation of diuron in paint layers. Monuron, dichloroaniline, 3-(3,4-dichlorophenyl)-1-formyl-1-methylurea, 3-(4-chloro-3-hydroxyphenyl)-1-1-dimethylurea or 3-(3-chloro-4-hydroxyphenyl)-1-1-dimethylurea, fenuron, several dimers, and other substances were detected after UV exposure of diuron and selected transformation products in aqueous solutions. The amounts of DCPMU and DCPU in the eluates were considerably lower than the amount of diuron. The amount of DCPMU was higher than the amount of DCPU, especially in eluates for the white paint film—as was also observed under natural weather conditions in experiment A. In another study [7], a considerable decrease of diuron after UV radiation was also observed in layers of the white and the red paint. This, together with the fact that the amounts of the two demethylation products were rather small, supports the assumption that several transformation products other than DCPMU and DCPU can be formed from diuron.

**Figure 4:**
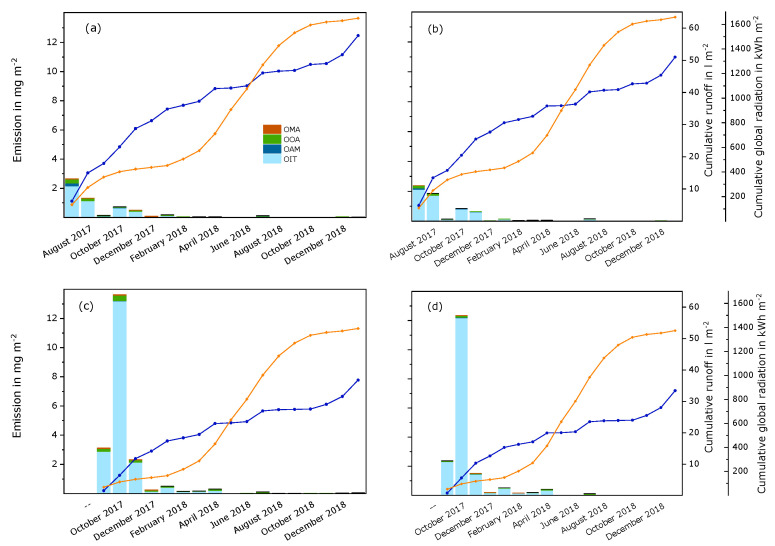
Monthly emissions of OIT and transformation products from a white and a red paint film during semi-field experiments that were started in July 2017 (experiment A) and September 2017 (experiment B). The columns represent the substances that were detected in runoff samples per month. The blue curves show the cumulated volume of runoff samples per surface area for each experiment. The orange curves show the cumulative global radiation during the experiment. (**a**) Experiment A, white paint; (**b**) experiment A, red paint; (**c**) experiment B, white paint; (**d**) experiment B, red paint.

OIT and transformation products. Emissions of OIT and degradation products were dominated by OIT and decreased with time (see Figure 4 and Supplementary Information SI S8). Similarly to diuron, the leached amount of OIT was especially high at the beginning of experiment B. This caused higher overall emissions during experiment B (18 mg m^−2^ to 21 mg m^−2^; about 8% to 10% of the initial amount) compared to experiment A (6 mg m^−2^ to 7 mg m^−2^; about 3% of the initial amount). The differences between the white and the red paint films seem to be neglectable. A very small increase of emissions was observed during July 2018. The concentrations of N-octyl oxamic acid (OOA) in the runoff samples were higher than the concentrations of octylamine (OAM) and N-octyl malonamic acid (OMA). OAM and OMA were detected only sporadically. Therefore, a course of emissions could only be observed for OOA. This secondary transformation product is supposed to be a degradation product of OMA [5]. Emissions of OOA increased mainly at the beginning of the experiments. Later, the curves flattened. The emitted amounts were similar in both experiments, but slightly higher in the white panels compared to the red panels. While OAM was detected at the beginning of experiment A, much lower emissions of this transformation product were observed at the beginning of experiment B.

The emissions of OIT and transformation products decreased with time, also in runoff from an acrylate and a silicone render [5]. During the second summer of the experiment, an increase of the emissions was observed. As for the paint films, concentrations in the runoff samples were higher for OOA than for OMA and OAM.

**Figure 5:**
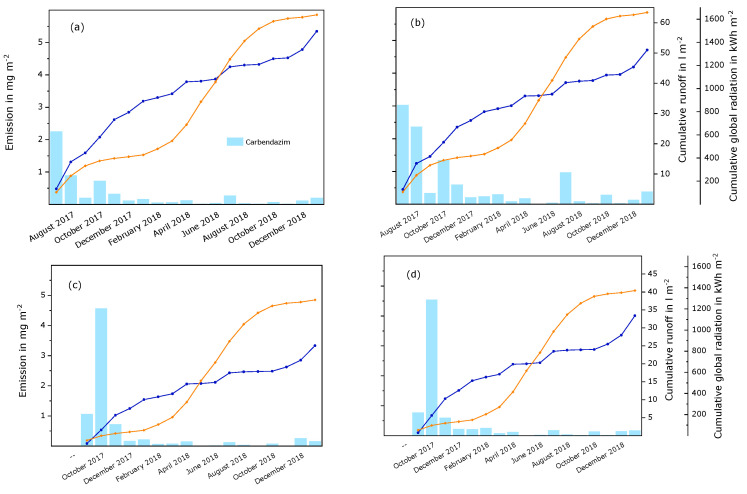
Monthly emissions of carbendazim from a white and a red paint film during semi-field experiments that were started in July 2017 (experiment A) and September 2017 (experiment B). The columns represent carbendazim that was detected in runoff samples per month. The blue curves show the cumulated volume of runoff samples per surface area for each experiment. The orange curves show the cumulative global radiation during the experiment. (**a**) Experiment A, white paint; (**b**) experiment A, red paint; (**c**) experiment B, white paint; (**d**) experiment B, red paint.

Carbendazim. As with the other investigated active substances, the emissions of carbendazim decreased with time (see Figure 5 and Supplementary Information SI 8). About 6 mg m^−2^ to 10 mg m^−2^ of carbendazim (about 3% to 4% of the initial amount) was emitted by the end of the experiments. Especially high emissions of carbendazim were observed during October 2017. However, in contrast to diuron and OIT, this did not result in higher overall emissions during experiment B. The highest amount of carbendazim was emitted from the red panel in experiment A. Under laboratory conditions, the amounts of carbendazim in eluates from test specimens were also higher for the red compared to the white paint film [8]. This was the case both for test specimens that were only exposed to water and for those that had water contact in combination with UV radiation. Lower emissions of carbendazim in the experiments that included UV radiation compared to leaching in the dark indicate that the substance can be transformed by photolysis. However, transformation products of carbendazim were not investigated during this study.

The phenomenon of relatively high emissions of the original active substances at the beginning of experiment B (started in September 2017) compared to experiment A (started in July 2017) indicates that less photolysis occurred in experiment B due to lower intensity of global radiation. Thus, the active substances were available for leaching. Large amounts of transformation products were observed for terbutryn at the beginning of experiment A. However, this was not the case for the other active substances, probably because relevant degradation products were not investigated.

Although not considered during this study, relative humidity and air temperature are presumed to affect reactions and transport processes in paint layers, too. Surfaces remain wet for a longer period of time at higher relative humidity. Transport of substances is facilitated within wet layers of paint. Transport processes are also accelerated at higher temperature. However, chemical reactions and evaporation of substances can also increase at higher temperatures and, in this way, reduce the amount of leached active substances.

### 3.4. Residues of Active Substances and Transformation Products in Test Specimens

Samples from upper, middle, and lower parts of the test panels were analyzed with regard to possible vertical transport of substances within the test panel during the period of outdoor exposure. The standard deviations of the residual amounts in the analyzed sections (*n* = 6) ranged between 10% and 20% for most of the analytes. The standard deviations were higher (up to 90%) for transformation products that were detected at low levels (TBSO, TBOH-DesE, and monuron). Small amounts of active substances and transformation products—usually less than 1% of the overall mass—were also detected in the uppermost 2 mm of the plywood, indicating that transport of substances into the panel also occurs. No influence of the position of the sections on the test panel was observed.

**Figure 6:**
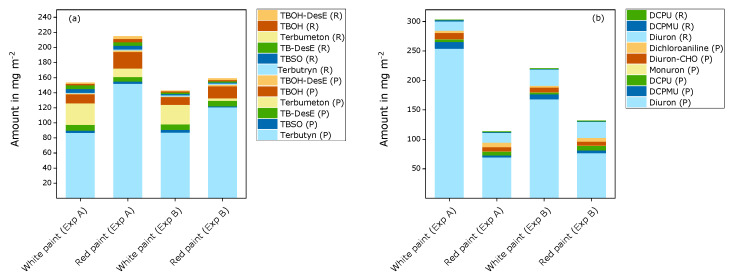
Mass balance of terbutryn and transformation products (**a**) and diuron and transformation products (**b**) in white and red test panels at the end of the semi-field experiment. The amounts of the transformation products are related to the initial amounts of the active substances, i.e., the initial amounts of transformed active substances were calculated and presented in the graphs. Data for the test panels (P): mean value of six sections; data for the runoff (R): sum of 83 (experiment A) and 69 (experiment B) runoff samples. Calculated original amounts for active substances: 230 mg m^−2^ (experiment A) and 218 mg m^−2^ (experiment B). Dichloroaniline, diuron-CHO, and monuron were only analyzed in the last 12 runoff samples.

Mass balances, including the data for the residual substances in the test panels and the cumulative amounts of leached substances in runoff samples, are demonstrated in Figure 6 (terbutryn and diuron) and Supplementary Information SI S9 (OIT and carbendazim). Related to the initial amounts, about 60% to 83% of terbutryn, 40% to 123% of diuron, 75% to 118% of OIT, and 72% to 88% of carbendazim were found in the test panels and the cumulated runoff, either as the original substance or as transformation products. It is to be noted that uncertainty in the calculation of the mass balances was relatively high, although standard deviations for parallel samples were within usual limits. However, data for runoff samples represent the sum of data on several substances in 69 and 83 samples, respectively. In addition, systematic errors cannot be excluded for the analysis of residues in the test panels since the recoveries for the substances in the aged paint films were not determined. In an earlier experiment, it became obvious that spiking of test specimens with mixtures of active substances and transformation products is rather complex and generates new sources of errors, even for fresh paint films [8]. In addition, the influence of aging of the paint layers cannot be reproduced reasonably. This might explain unexpected gaps in the mass balances for terbutryn, although it is assumed that the most important transformation products were considered in this study. Further degradation of the transformation products may be possible, however. As a consequence, semi-quantitative comparison of the results for the different test panels seems to be more justified than direct comparison of the analytical data.

The residues of the active substances and transformation products in the test panels were higher than the cumulated amounts of leached substances in the runoff, with higher amounts of the active substances than of the transformation products. In the runoff samples, the amounts of diuron and OIT were higher than the amounts of transformation products, while for terbutryn, the runoff samples were dominated by the transformation products. It is remarkable that the amounts of terbutryn, OIT, and carbendazim were highest in the test panel of the red paint for experiment A. Here, accidentally, two layers of paint without film preservatives were applied first and then covered with two layers of paint including the active substances. Part of the active substances may have been transferred to the lower paint layers and, in this way, better protected from photolysis and leaching.

In most cases, the observed patterns of substances appeared to be similar for each paint. For transformation products of terbutryn in the test panels, higher amounts of terbumeton and slightly lower amounts of TBOH were detected in the white panels compared to the red panels. Terbumeton was not detected in runoff samples. This is similar to observations under laboratory conditions, where terbumeton was also detected in extracts from the paint layers, but not in eluents from leaching tests [8]. It is not clear whether this observation was caused by an analytical artefact, given that it is expected that terbumeton can be leached from the paint films, as are the other transformation products. The relative amount of transformation products compared to the remaining terbutryn was higher when coated glass test specimens were exposed to UV radiation in a weathering device and intermittent water contact under the applied laboratory conditions [8].

The amounts of diuron and transformation products that were detected up to the end of the outdoor exposure were considerably lower in the red panels than in the white panels. This can be interpreted as an indication of faster degradation or additional transformation pathways of diuron in the red paint film. The concentration of the primary demethylation product DCPMU was higher in the white panels than in the red panels, while the concentration of the secondary demethylation product DCPU was slightly higher in the red panels. This is another indication of the different relevance or velocity of transformation pathways in paints containing different pigments. Under laboratory conditions, the amounts of diuron and transformation products decreased rapidly in the paint test specimens under the influence of UV radiation. Given that this decrease was not explained by losses due to leaching, it is interpreted as an indication of additional transformation products that have not been investigated [8].

Fast degradation of OIT was observed under laboratory [7] and outdoor conditions [5]. This also applies to the transformation products. In addition, it is assumed that evaporation of octylamine [OAM] or binding of degradation products to the polymer matrix affect mass balances of OIT [5]. Only very small amounts of OIT transformation products were detected, suggesting that fast degradation of these transformation products also occurred during this study.

Differences between the paints were not observed for carbendazim mass balances, despite the relatively high amount of carbendazim in the red panel of experiment A.

## 4. Conclusions

Transformation and leaching processes of the investigated active substances from paint films depend on water contact with the surfaces, as well as energy input due to global radiation. However, the actual influence of these parameters can vary for different substances. Transformation processes and transport of substances to the surface of a paint layer take time and are influenced by different weather factors, which change during the seasons and from one year to the next. Therefore, different processes can take place preferentially at different times. For this reason, a proactive comprehensive assessment of biocide emissions under true natural conditions is hardly possible, and suitable simplifications are required. However, the prediction of emissions in relation to the expected amounts of rain—or driving rain in case of vertical constructions—is a very rough estimate that does not consider possible transformation reactions. As far as available, results from laboratory studies on transformation processes are a good basis for predicting transformation products that can be expected under natural weather conditions.

Transformation processes depend not only on weather conditions, but also on the chemical environment, e.g., binders and pigments in paint films. Therefore, it is advisable to investigate complete products of different compositions rather than solutions of the substances of interest.

In addition to strategies to minimize environmental impacts of biocidal active substances, it is also necessary to know their transformation products in order to obtain an overview of the substances in construction products that enter the environment. However, to assess the environmental impacts of transformation products, their environmental characteristics, i.e., the ecotoxicity and the fate in the environment, must be known. So far, the required data are only available for some transformation products. Evidence of transformation products can be reason to generate the required data.

During the present study, transformation products occurred mainly during seasons of high global radiation and in subsequent periods with relatively high amounts of driving rain. Such conditions are expected to be more frequent for the experimental site region due to climate change. If this occurs, environmentally harmful transformation products may have a higher damage potential.

The observations that larger amounts of transformation products are formed at higher global radiation and that the leaching of substances increases during long-lasting phases with humid weather, can be useful for data from monitoring programs that are typically collected only for specific time points. These snapshots, however, do not allow conclusive statements regarding emission progress and transformation processes. As an example, the occurrence of transformation products can be an indicator for the presence of terbutryn.

Thus, further investigation of the transformation of diuron and carbendazim in paint films is required to provide a complete understanding of the transformation products of these substances.

This study has yielded the following answers to the questions raised at the beginning: (1) transformation products occurring under natural weather conditions have been described and indications of further transformation products have been observed, (2) precipitation and global radiation act in combination on release processes of biocides from paint films, (3) transformation processes can be influenced by pigments under natural weather conditions, and (4) certain basic observations are similar in the laboratory and in the field.

## Figures and Tables

**Table 1:** Analyzed active substances (bold) and transformation products.

Substance	Code	CAS
**Terbutryn**		886-50-0
Desethyl terbutryn	TB-DesE	30125-65-6
2-Hydroxy terbutryn	TBOH	66753-07-9
Desethyl-2-hydroxy-terbutryn	TBOH-DesE	66753-06-8
Terbutryn sulfoxide	TBSO	82985-33-9
Terbumeton		33693-04-8
**Diuron**		330-54-1
1-(3,4-Dichlorophenyl-3)-methyl urea	DCPMU	3567-62-2
1-(3,4-Dichlorophenyl)-urea	DCPU	2327-02-8
3-(3,4-Dichlorophenyl)-1- formyl-1-methylurea	Diuron-CHO	76409-92-2
Monuron	Diuron-DesCl	150-68-5
Dichloraniline	DCA	95-76-1
**Carbendazim**		10605-21-7
**Octylisothiazolinone**	OIT	26530-20-1
Octylamine	OAM	111-86-4
N-Octyl oxamic acid	OOA	3151-48-2
N-Octyl malonamic acid	OMA	3151-49-3

**Table 2:** Total precipitation, driving rain, global radiation, and runoff from the test panels during the experiments.

Experiment	Duration	Precipitation	Driving Rain *	Global Radiation	Runoff from White Panel	Runoff from Red Panel
	Months	L m^−2^	L m^−2^	kWh m^−2^	L	L
Experiment A	18.5	995	146	1658	58	51
Experiment B	16.5	677	129	1374	36	33

* calculated according to EN ISO 15927-3 [21].

**Table 3:** Rough description of precipitation and global radiation during the seasons of the semi-field experiment. Graphs of the weather conditions during the experiments are given in Supplementary Information SI S5.

Phase	Season	Precipitation	Global Radiation
1	summer 2017	high	moderate
2	autumn 2017	high	moderate
3	winter 2017/2018	high (despite of February 2018)	low
4	spring 2018	high (despite of May 2018)	moderate
5	summer 2018	low (despite of one heavy rain event in July 2018)	high
6	autumn 2018	low	moderate
7	winter 2018/2019	high	low

## Data Availability

The data presented in this study are available from the authors.

## Supplementary Materials

The following supporting information can be downloaded at: https://www.mdpi.com/article/10.3390/ma15207368/s1, Supplementary Information SI S1: Composition of the investigated paints, Supplementary Information SI S2: Experiments, Supplementary Information SI S3: Analytical methods, Supplementary Information SI S4: Analysed active substances and transformation products, Supplementary Information SI S5: Weather conditions during the experiments, Supplementary Information SI S6: Surface temperatures on white and red paints, Supplementary Information SI S7: Concentration of active substances and transformation products in runoff-samples, Supplementary Information SI S8: Emission curves, Supplementary Information SI S9: Mass balances. Refs. [8,20,21,22,23,24,25] can be found in Supplementary Materials.

## References

[1] Burkhardt M., Zuleeg S., Vonbank R., Bester K., Carmeliet J., Boller M., Wangler T. (2012). Leaching of biocides from facades under natural weather conditions. Environ. Sci. Technol..

[2] Bollmann U.E., Minelgaite G., Schlüsener M., Ternes T., Vollertsen J., Bester K. (2016). Leaching of terbutryn and its photodegradation products from artificial walls under natural weather conditions. Environ. Sci. Technol..

[3] Schoknecht U., Mathies H., Wegner R., Uhlig S., Baldauf H., Colson B. (2016). Emissions of Material Preservatives into the Environment—Realistic Estimation of Environmental Risks through the Improved Characterisation of the Leaching of Biocides from Treated Materials Used Outdoors. UBA-Texte 22/2016.

[4] Schoknecht U., Mathies H., Wegner R. (2016). Biocide leaching during field experiments on treated articles. Environ. Sci. Eur..

[5] Bollmann U.E., Minelgaite G., Schlüsener M., Ternes T.A., Vollertsen J., Bester K. (2017). Photodegradation of octylisothiazolinone and semi-field emissions from facade coatings. Sci. Rep..

[6] Hensen B., Lange J., Jackisch N., Zieger F., Olsson O., Kümmerer K. (2018). Entry of biocides and their transformation products into groundwater via urban stormwater infiltration systems. Water Res..

[7] Urbanczyk M.M., Bester K., Borho N., Schoknecht U., Bollmann U.E. (2019). Influence of pigments on phototransformation of biocides in paints. J. Hazard. Mater..

[8] Schoknecht U., Mathies H., Lisec J. (2021). Leaching and transformation of film preservatives in paints induced by combined exposure to ultraviolet radiation and water contact under controlled laboratory conditions. Water.

[9] Linke F., Olsson O., Preusser F., Kümmerer K., Schnarr L., Bork M., Lange J. (2021). Sources and pathways of biocides and their transformation products in urban storm water infrastructure of a 2 ha urban district. Hydrol. Earth Syst. Sci..

[10] Wicke D., Tatis-Muvdi R., Rouault P., Zerball-van Baar P., Dünnbier U., Rohr M., Burkhardt M. (2022). Emissions from building materials—A threat to the environment?. Water.

[11] European Parliament and Council, Regulation (EU) No 528/2012 Concerning the Making Available on the Market and Use of Biocidal Products. https://echa.europa.eu/regulations/biocidal-products-regulation/legislation.

[12] Tanaka F.S., Hoffer B.L., Wien R.G. (1986). Photolysis of 3-(3,4-dichlorophenyl)-1,1-dimethylurea (diuron) in dilute aqueous solution. Toxicol. Environ. Chem..

[13] Jirkovský J., Faure V., Boule P. (1997). Photolysis of diuron. Pestic. Sci..

[14] Assessment Report: Carbendazim Product-Type 7 (Film Preservative) and 10 (Construction Material Preservative), November 2019. https://echa.europa.eu/documents/10162/ee122398-131d-a017-07b5-499c1332de0f.

[15] Bollmann U.E., Vollertsen J., Carmeliet J., Bester K. (2014). Dynamics of biocide emissions from buildings in a suburban stormwater catchment—Concentrations, mass loads and emission processes. Water Res..

[16] Junginger T., Payraudeau S., Imfeld G. (2022). Transformation and stable isotope fractionation of the urban biocide terbutryn during biodegradation, photodegradation and abiotic hydrolysis. Chemosphere.

[17] Paijens C., Bressy A., Frère B., Moilleron R. (2020). Biocide emissions from building materials during wet weather: Identification of substances, mechanism of release and transfer to the aquatic environment. Environ. Sci. Pollut. Res..

[18] Uhlig S., Colson B., Schoknecht U. (2019). A mathematical approach for the analysis of data obtained from the monitoring of biocides leached from treated materials exposed to outdoor conditions. Chemosphere.

[19] ECHA Website Information on Biocides. https://echa.europa.eu/information-on-chemicals/biocidal-active-substances.

[20] Deutscher Wetterdienst. https://www.dwd.de/DE/leistungen/klimadatendeuschland/vielj_mittelwerte.html.

[21] (2009). Hygrothermal Performance of Buildings—Calculation and Presentation of Climatic Data—Part 3: Calculation of Driving Rain Index for Vertical Surfaces from Hourly Wind and Rain Data.

[22] Chemspider. http://www.chemspider.com/.

[23] Pubchem. https://pubchem.ncbi.nlm.nih.gov/compound/Octylcarbamoyl_formic-acid.

[24] Umweltbundesamt. https://www.umweltbundesamt.de/sites/default/files/medien/2875/dokumente/umweltqualitaetsnormen_des_chemischen_zustands_0.pdf.

[25] Wünnemann H., Weiß K., Arndt D., Baumann M., Weiß R., Ferling H., Scholz-Göppel K., Bucher K., Feick Ch Hartmann G., Kitzing P. (2020). Umweltqualitätsnormen für Binnengewässer.

