# Supplementary material for: The Impact of Weather Conditions on Biocides in Paints
Source: Materials (Basel). 2022 Oct 20;15(20):7368. doi: 10.3390/ma15207368 (PMC9609615; doi:10.3390/ma15207368)
Supplement: Supplementary file 1 [file materials-15-07368-s001.zip › materials-1964234-supplementary.pdf]

The impact of weather conditions on biocides in paints

Supplementary Materials

Ute Schoknecht, Helena Mathies*

Federal Institute for Materials Research and Testing, Unter den Eichen 87, 12205 Berlin

SI 1 Composition of the investigated paints

Ingredient	Red paint<br>Wt.-%	White paint<br>Wt.-%
Water	17.32	17.32
Cellulose ether	0.19	0.19
Sodium hydroxide solution 20%	0.19	0.19
Na-Polyacrylate 35%	0.28	0.28
Polymer defoaming agent	0.28	0.28
Methylisothiazolinone/benzisothiazolinone	0.19	0.19
TiO_2 chloride type, Color index: P.W. 6	0.00	9.21
Iron oxide red / Fe_2O_3 / color index P.R. 101	9.21	0.00
Mica D: 50 38 µ	4.46	4.46
$CaCO_3$ D: 50 5 µ	13.86	13.86
$CaCO_3$ D: 50 15 µ	22.77	22.77
Styrene acrylate 50 % MFT ca. 20 Grad	27.72	27.72
Silicone resin emulsion modified polysiloxane 55%	0.99	0.99
Acrylate thickener 25%	0.28	0.28
Dipropylene glycol n-butylether	1.29	1.29
Dispersion containing 25% terbutryn [a]	0.20	0.20
Dispersion containing 50% diuron [a]	0.10	0.10
Dispersion containing 8% OIT [a]	0.62	0.62
Dispersion containing 50% carbendazim [a]	0.10	0.11
Sum	100	100

[a] biocides dispersions including surfactants

SI 2: Experiments

Experiment	Application of paint	Dimension (height by widht) in mm · mm	Area in m²	Amount of wet paint with biocide in g m^{-2}	Start	End	Months (total)	Active substance	Initial amount of active substance (calculated*) in mg m^{-2}
A - white	June 2017	760 · 720	0.547	461	11th July 2017	31st January 2019	18.5	Carbendazim	231
								Diuron	231
								Terbutryn	231
								OIT	231
A - red	June and July 2017	760 · 720	0.547	461	11th July 2017	31st January 2019	18.5	Carbendazim	231
								Diuron	231
								Terbutryn	231
								OIT	231
B - white	September 2017	760 · 760	0.578	437	13th September 2017	31st January 2019	16.5	Carbendazim	219
								Diuron	219
								Terbutryn	219
								OIT	219
B - red	September 2017	760 · 760	0.578	437	13th September 2017	31st January 2019	16.5	Carbendazim	219
								Diuron	219
								Terbutryn	219
								OIT	219

* content of active substances in the wet pain according to the recipe: 0.05 %

SI 3: Analytical methods

LC-MS Analysis

LC-System:	Agilent 1100 Series
Detector:	Agilent 6130 Series Quadrupol
Column:	Phenomenex Synergi^{TM}, 4 µm Hydro-RP 80 Å, 150 mm x 2 mm
Column temperature:	30 °C
Eluent:	A: 0.2% acetic acid in water (Millipore quality)
	B: acetonitrile, LC-MS quality (Chemsolute)
Flow:	0.5 ml min^{-1}
Injection volume:	10 µl
Gradient:	

Time	Solvent A	Solvent B
min	%	%
1	90	10
4	50	50
10	25	75
11	10	90
11.5	90	10
15	90	10

Quadrupole temperature:	100 °C
MSD spray chamber:	API-ES
Drying gas flow:	12 l min^{-1} nitrogen
Nebulizer pressure:	40 psig
Drying gas temperature:	300 °C
Capillary voltage:	positive mode: 1500 V, negative mode: 1500 V

LC-MS Analysis for OIT transformation products (N-octyl oxamic acid, N-octyl malonamic acid)

LC-System:	Agilent 1100 Series
Detector:	Agilent 6130 Series Quadrupol
Column:	
Column temperature:	25 °C
Eluent:	A: 0.2% formic acid in water (Millipore quality)
	B: 0.2 % formic acid in methanol, LC-MS quality (Chemsolute)
Flow:	0.25 ml min^{-1}
Injection volume:	10 µl

Gradient:

Time	Solvent A	Solvent B
min	%	%
1	100	0
4.5	0	100
7.5	0	100
7.8	100	0
15	100	0

Quadrupole temperature: 100 °C
MSD spray chamber: API-ES
Drying gas flow: 12 l min^{-1} nitrogen
Nebulizer pressure: 40 psig
Drying gas temperature : 300 °C
Capillary voltage: positive mode: 1500 V, negative mode: 1500 V

Signals

Substance	Code	Retention time	Mode	Signal M+1	Signal M-1	Calibration range
		min		m/z	m/z	mg l^{-1}
Terbutryn		8.93	positive	242		0.0004 - 0.2
Terbumeton		7.04	positive	226		0.001 - 0.5
2-Hydroxy terbutryn	TBOH	5.13	positive	212		0.002 - 1.0
Desethyl-2-hydroxy-terbutryn	TBOH-DesE	1.54	positive	184		0.004 - 2.0
Desethyl terbutryn	TB-DesE	7.17	positive	214		0.002 - 1.0
Terbutryn sulfoxide	TBSO	7.16	positive	258		0.002 - 0.5
Diuron		7.95	positive	233		0.002 - 2.0
1-(3,4-Dichlorphenyl-3) -methyl urea	DCPMU	7.49	negative		218	0.002 - 0.2
1-(3,4-Dichlor-phenyl)-urea	DCPU	7.04	negative		203	0.001 - 0.05
Dichloranilin		7.08	positive	162		0.005 - 5
3-(3,4-dichloro-phenyl)-1- formyl-1-methylurea	Diuron-CHO	8.69	negative		245	0.002 – 0.2
Monuron		7.02	positive	199		0.002 - 0.10
Carbendazim		3.70	positive	192		0.002 - 2.0
Octylisothiazolinone	OIT	10.28	positive	214		0.005 - 1.0
Octylamine	OAM	5.38	positive	130		0.005 – 1.0
N-Octyl oxamic acid	OOA	8.58	negative		200	0.001 – 0.1
N-Octyl malonamic acid	OMA	8.56	negative		214	0.001 – 0.1

A matrix signal was observed for the same retention time and m/z as for TB-DesE.

Standards

Substance	Code	CAS	Supplier	Purity
Terbutryn		886-50-0	Dr. Ehrenstorfer	99.0%
Terbumeton		33693-04-8	Sigma-Aldrich	99.2%
2-Hydroxy terbutryn	TBOH	66753-07-9	Sigma-Aldrich	98.6%
Desethyl-2-hydroxy-terbutryn	TBOH-DesE	66753-06-89	Neochema	10 µg ml^{-1}
Desethyl terbutryn	TB-DesE	30125-65-6	Neochema	99.8%
Terbutryn sulfoxide	TBSO	82985-33-9	Neochema	98.5%
Diuron		330-54-1	Sigma-Aldrich	> 98.0%
1-(3,4-Dichlorophenyl-3) -methyl urea	DCPMU	3567-62-2	Dr. Ehrenstorfer	99.0%
1-(3,4-Dichlorophenyl)-urea	DCPU	2327-02-8	Neochema	100 µg ml^{-1}
Dichloraniline	DCA	95-76-1	Ultra Scientific	≥ 98.0%
3-(3,4-dichlorophenyl)-1-formyl-1-methylurea	Diuron-CHO	76409-92-2	Sigma-Aldrich	contained DCPMU
Monuron		150-68-5	Sigma-Aldrich	99.9%
Carbendazim		10605-21-7	Sigma-Aldrich	99.5%
Octylisothiazolinone	OIT	26530-20-1	Dr. Ehrenstorfer	99.5%
Octylamine	OAM	111-86-4	Sigma-Aldrich	99.5%
N-Octyl oxamic acid	OOA	3151-48-2	ChemSpace	95.0%
N-Octyl malonamic acid	OMA	3151-49-3	ChemSpace	95.0%

SI 4 Analysed active substances and transformation products

A more comprehensive list of transformation products of terbutryn and diuron can be found in SI of [1].

No	Substance (IUPAC name)	Code	CAS	Mass in g mol^{-1}	Molecular formula	Structure
1	Terbutryn<br>((4E)-4-(Ethylimino)-N-(2-methyl-2-propanyl)-6-(methylsulfanyl)-1,4-dihydro-1,3,5-triazin-2-amine)		886-50-0	241.356	$C_{10}H_{19}N_5S$	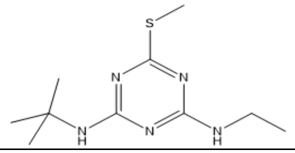
2	Desethyl-terbutryn<br>(4-Imino-N-(2-methyl-2-propanyl)-6-(methylsulfanyl)-1,4-dihydro-1,3,5-triazine-2-amine	TB-DesE	30125-65-6	213.303	$C_8H_{15}N_5S$	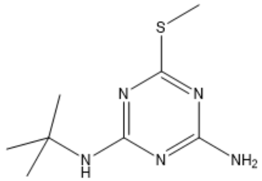
3	2-Hydroxy-terbutryn<br>(4-(Ethylamino)-6-[(2-methyl-2-propanyl)amino]-1,3,5-triazin-2(5H)-one)	TBOH	66753-07-9	211.264	$C_9H_{17}N_5O$	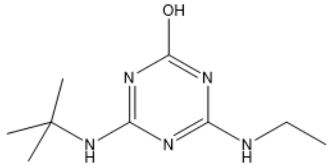
4	Desethyl-2-hydroxy-terbutryn<br>(4-Amino-6-[(2-methyl-2-propanyl)amino]-1,3,5-triazin-2(5H)-one)	TBOH-DesE	66753-06-8	183.211	$C_7H_{13}N_5O$	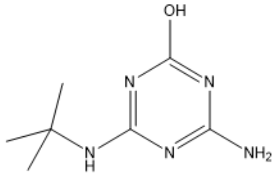
5	Terbutrynsulfoxid<br>(N-Ethyl-N'-(2-methyl-2-propanyl)-6-(methylsulfinyl)-1,3,5-triazine-2,4-diamine)	TBSO	82985-33-9	257.356	$C_{10}H_{19}N_5OS$	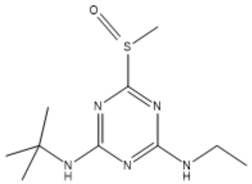

6	Terbumeton<br>(4E)-4-(Ethylimino)-6-methoxy-N-(2-methyl-2-propanyl)-1,4-dihydro-1,3,5-triazine-2-amine)	Terbumeton	33693-04-8	225.291	$C_{10}H_{19}N_5O$	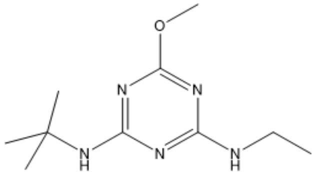
7	Diuron<br>(3-(3,4-Dichlorophenyl)-1,1-dimethylurea)		330-54-1	233.094	$C_9H_{10}Cl_2N_2O$	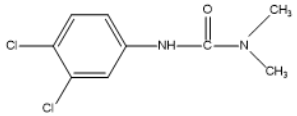
8	Desmethyl-diuron<br>(1-(3,4-Dichlorophenyl)-3-methylurea)	DCPMU	3567-62-2	219.068	$C_8H_8Cl_2N_2O$	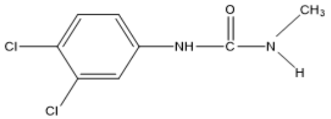
9	Desdimethyl-diuron<br>(1-(3,4-Dichlorophenyl)urea)	DCPU	2327-02-8	205.041	$C_7H_6Cl_2N_2O$	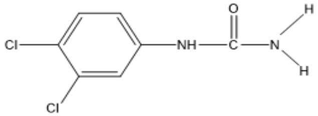
10	3-(3,4-Dichlorophenyl)-1-formyl-1-methylurea	Diuron-CHO	76409-92-2	247.078	$C_9H_8Cl_2N_2O_2$	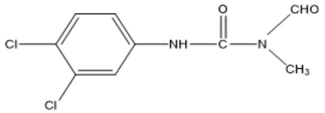
11	Monuron<br>3-(4-Chlorophenyl)-1,1-dimethylurea	Diuron-DesCl	150-68-5	198.649	$C_9H_{11}ClN_2O$	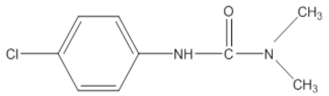
12	Dichloroaniline<br>(3,4-Dichloraniline)	DCA	95-76-1	162.017	$C_6H_5Cl_2N$	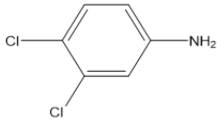
13	Carbendazim<br>(Methyl-benzimidazol-2-yl-carbamate)		10605-21-7	191.187	$C_9H_9N_3O_2$	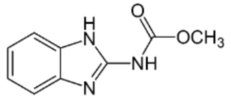

14	Octylisothiazolinone (2-Octyl-1,2-thiazol-3(2H)-one	OIT	26530-20-1	213.340	$C_{11}H_{19}NOS$	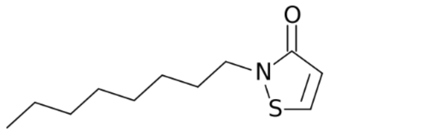
15	Octylamine (1-Octanamine)	OAM	111-86-4	129.243	$C_8H_{19}N$	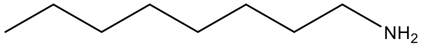
16	N-Octyl oxamic acid	OOA	3151-48-2*	201.1365*	$C_{10}H_{19}NO_3$	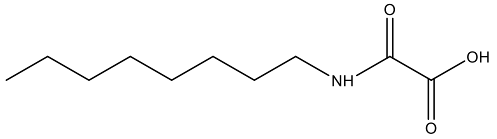
17	N-Octyl malonamic acid (3-(Octylamino)-3-oxopropanoic acid)	OMA	3151-49-3	215.289	$C_{11}H_{21}NO_3$	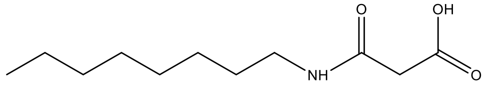

Mass data for originate from Chemspider [2] despite of OOA (data from Pubchem [3])

SI 5: Weather conditions during the experiments

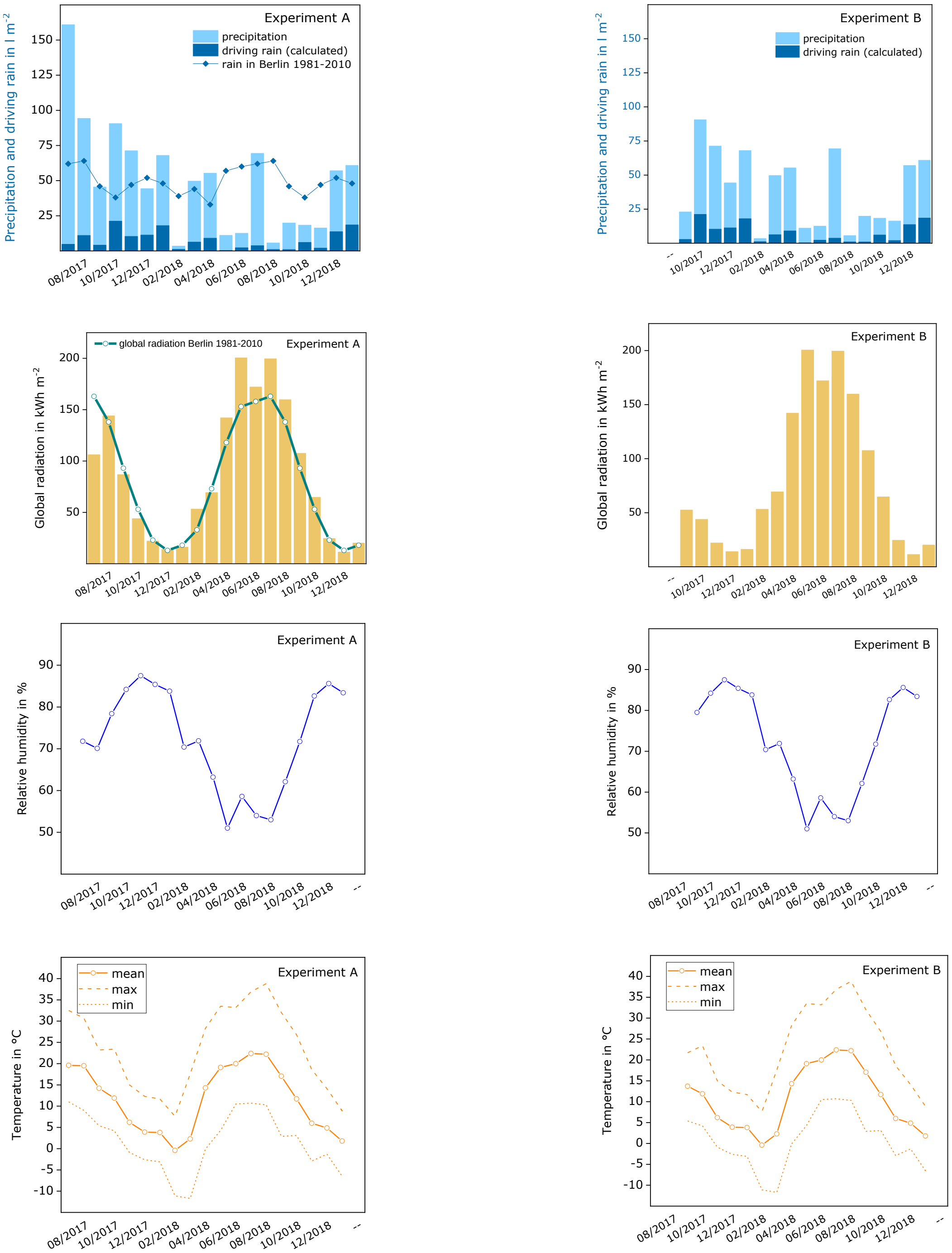

Figure S1. Monthly summarized weather data. Columns and data points for the first month of each experiment include only data from the start of the experiments. The graphs show total amounts of precipitation and calculated driving rain (top row), total amounts of global radiation (second row), mean values of relative humidity (third row), and mean as well as minimum and maximum temperatures (bottom row). Mean data for 1981 to 2010 measured in Berlin-Dahlem [4] are included for precipitation and global radiation. Driving rain was calculated according to EN ISO 15927-3 [5].

SI 6: Surface temperatures on white and red paints

The surface temperatures on the test panels were measured by an infrared thermometer (EVENTEK) at different positions of the test panels. The air temperatures were obtained from the DWD (Deutscher Wetterdienst) for the test location.

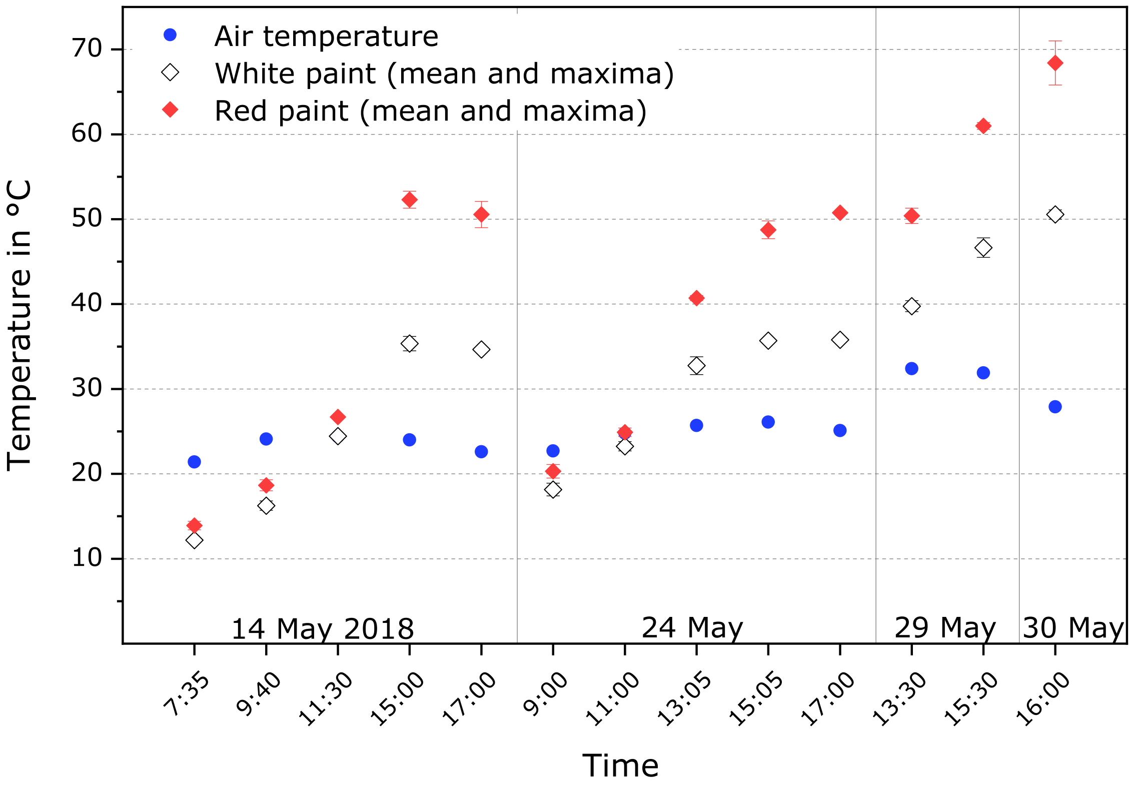

Figure S2. Surface temperatures on the test panels.

SI 7: Concentration of active substances and transformation products in runoff-samples

The following graphs show the range of the concentrations of the different analytes including median and maximum values. The concentrations of several samples were below the lowest standard concentration. However, the signals were well evaluable.

Experiment A, Terbutryn and TPs

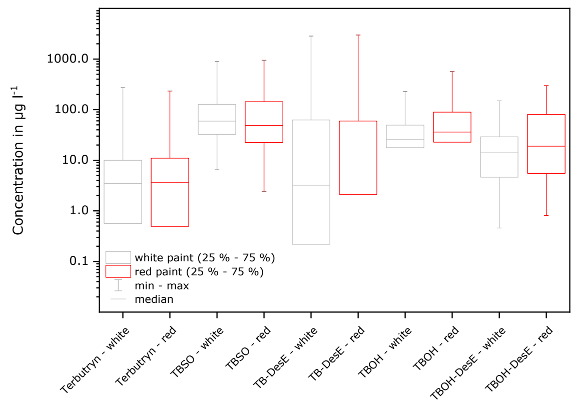

Experiment B, Terbutryn and TPs

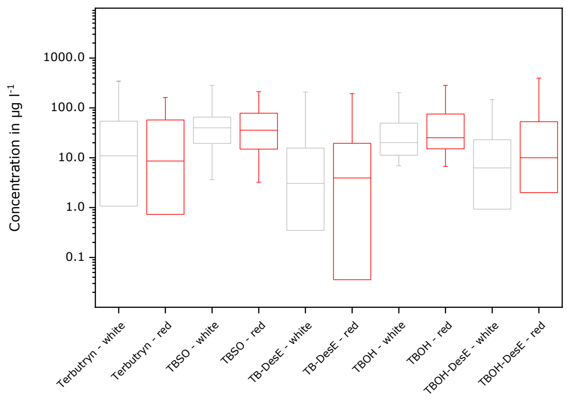

Experiment A, Diuron and TPs

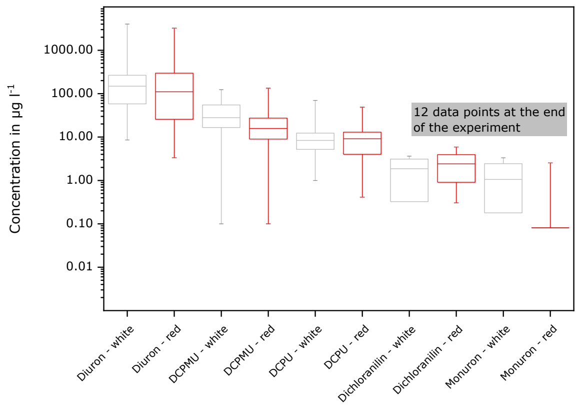

Experiment B, Diuron and TPs

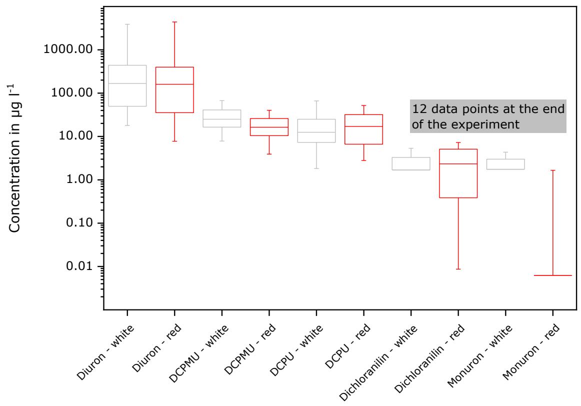

Experiment A, OIT and TPs

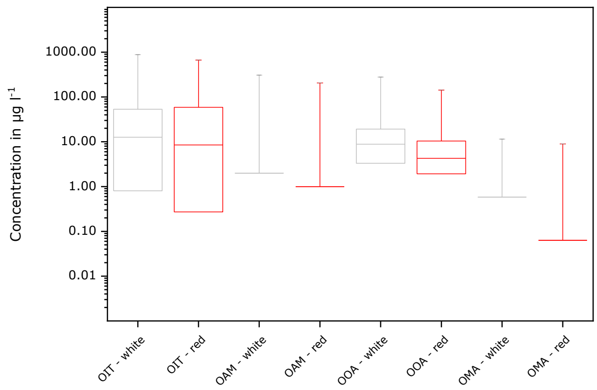

Experiment B, OIT and TPs

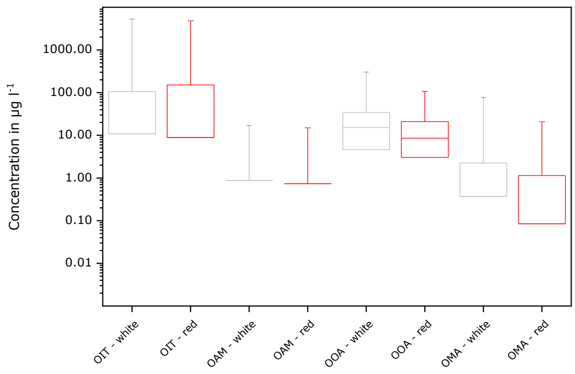

Experiment A and B, Carbendazim

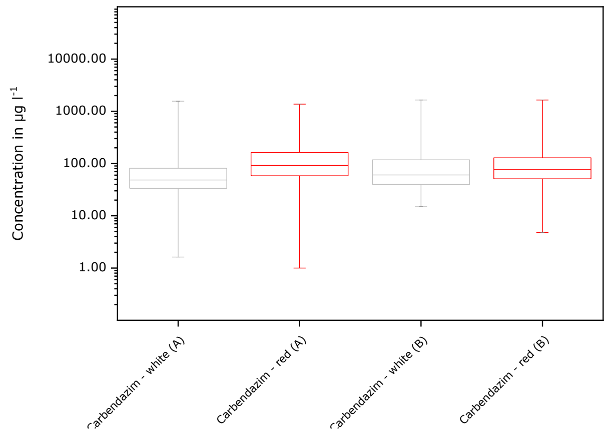

Figure S3. Concentration of active substances and transformation products in runoff samples.

Environmental quality standards (Umweltqualitätsnormen UQN, JD-UQN: annual average, ZHK-UQN: maximum allowable concentration) have been defined or proposed for a few substances in Germany. Concentrations of these substances exceed these standards in many runoff samples (see Table SI 7-1).

Table S1. Environmental quality standards.

Substance	JD-UQN	ZHK-UQN	Number of samples above ZHK-UQN			
			Experiment A<br>83 samples		Experiment B<br>69 samples	
			White paint	Red paint	White paint	Red paint
Terbutryn *	0.065 µg l^{-1}	0.34 µg l^{-1}	64	64	56	53
TB-DesE**	0.22 µg l^{-1}	0.77 µg l^{-1}	55	45	47	46
Diuron*	0.2 µg l^{-1}	1.8 µg l^{-1}	83	83	69	69
			Number of samples above JD-UQN			
TBSO**	30 µg l^{-1}		64	54	40	39

*defined values: Umweltbundesamt [6]

**proposed values [7]

SI 8: Emission curves

The data for transformation products in the graphs are related to the original amount of the biocidal active substances, i.e., the data show the amount of the biocidal active substance that is transformed to a certain product.

<u>Terbutryn and transformation products in runoff samples</u>

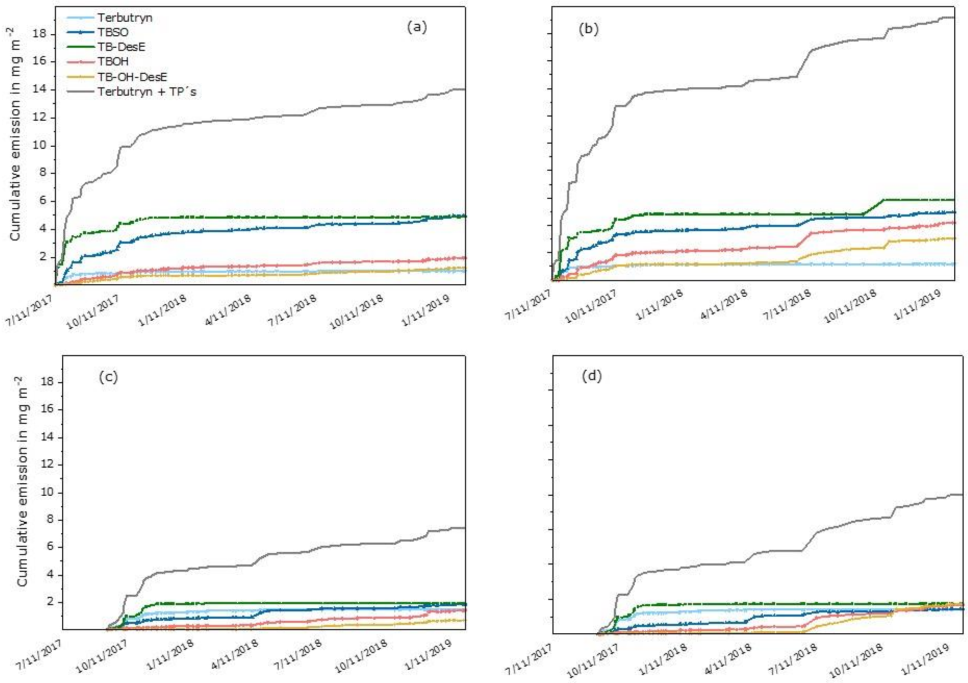

Figure S4. Emission curves of terbutryn and transformation products, (a) experiment A, white paint, (b) experiment A, red paint, (c) experiment B, white paint, (d) experiment B, red paint.

Diuron and transformation products in runoff samples

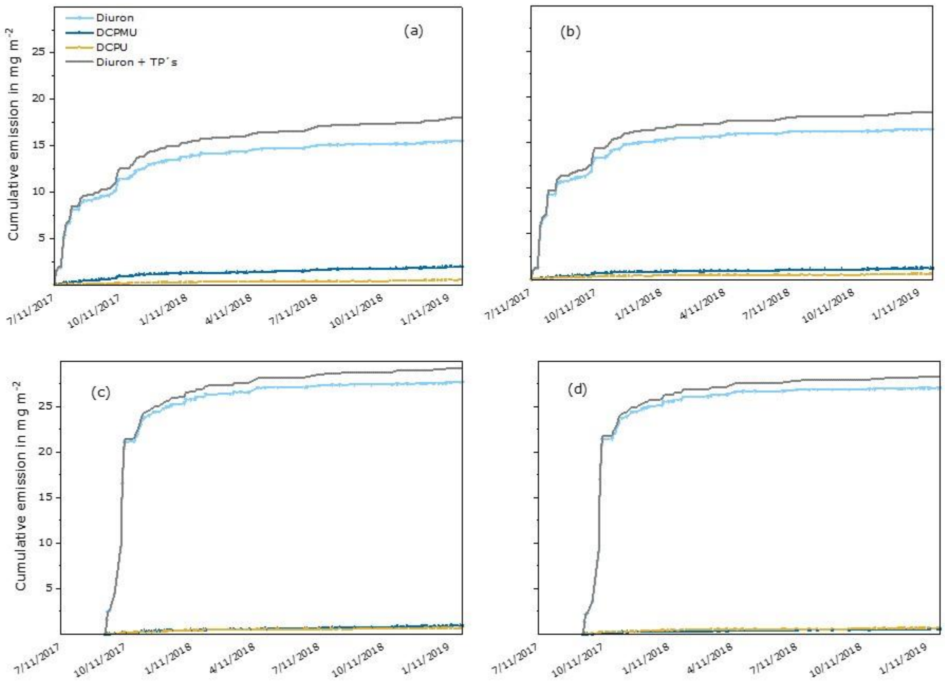

Enlarged version to visualize emission courses of DCPMU and DCPU

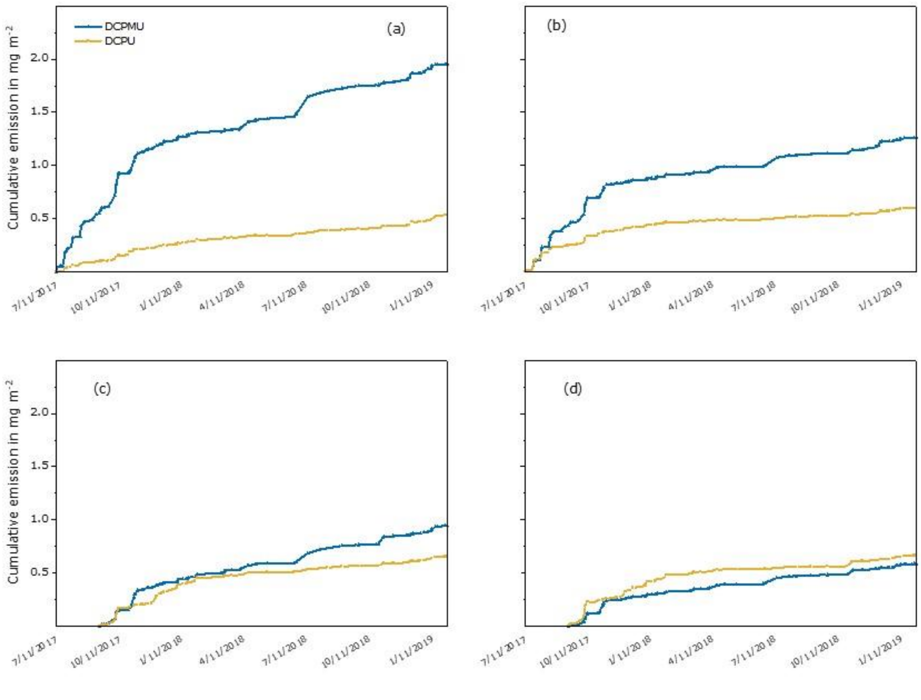

Figure S5. Emission curves of diuron and transformation products, (a) experiment A, white paint, (b) experiment A, red paint, (c) experiment B, white paint, (d) experiment B, red paint.

OIT and transformation products in runoff samples

Five runoff samples of experiment A, collected between 25 September and 23 October 2018, were not analyzed for OOA and OMA. The missing values were estimated by using the mean concentrations of five samples before and five sample after the gap.

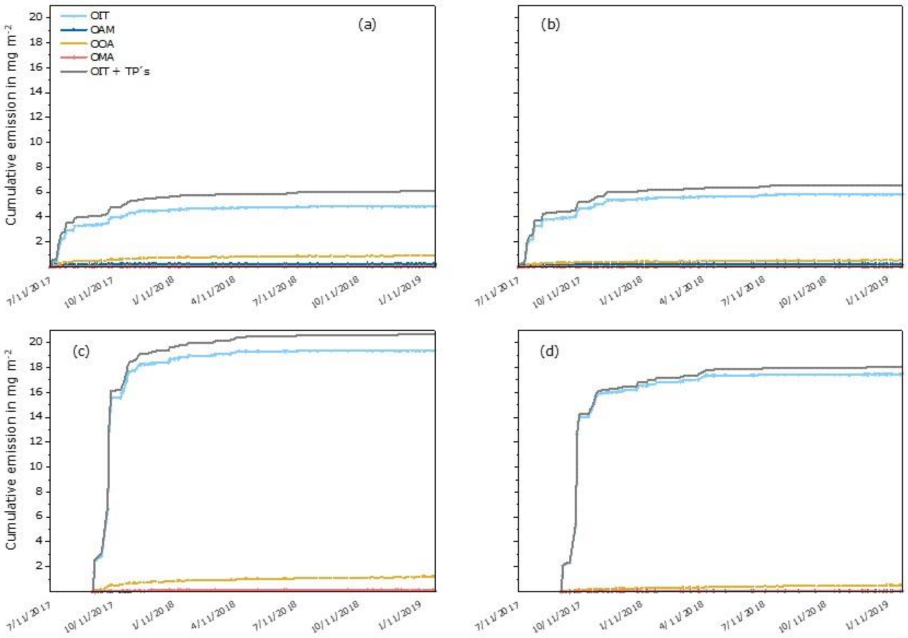

Enlarged version to visualize emission courses of OAM, OOA and OMA

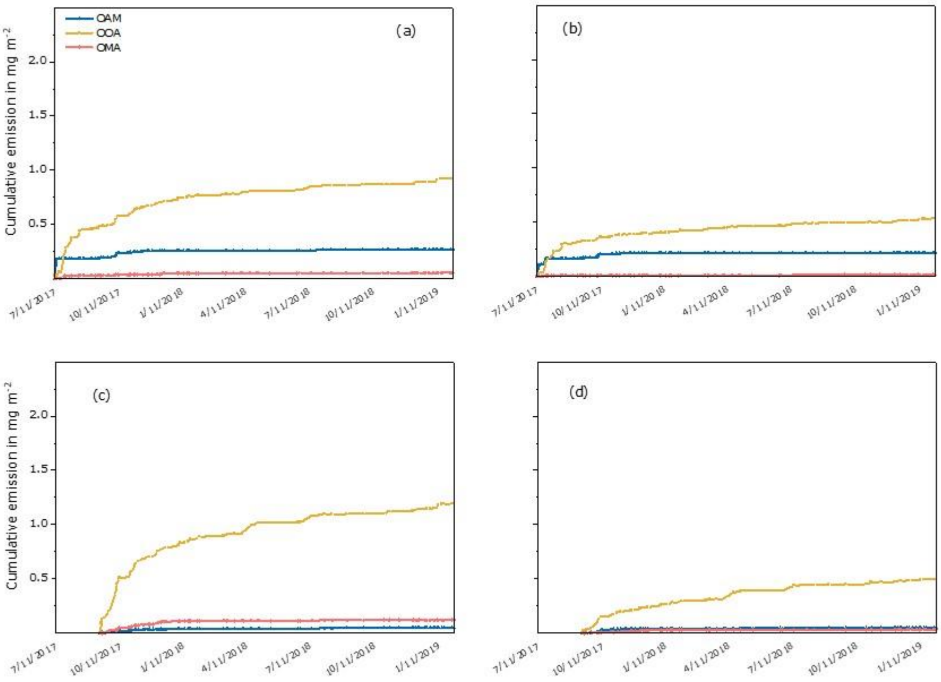

Figure S6. Emission curves of OIT and transformation products, (a) experiment A, white paint, (b) experiment A, red paint, (c) experiment B, white paint, (d) experiment B, red paint .

Carbendazim in runoff samples

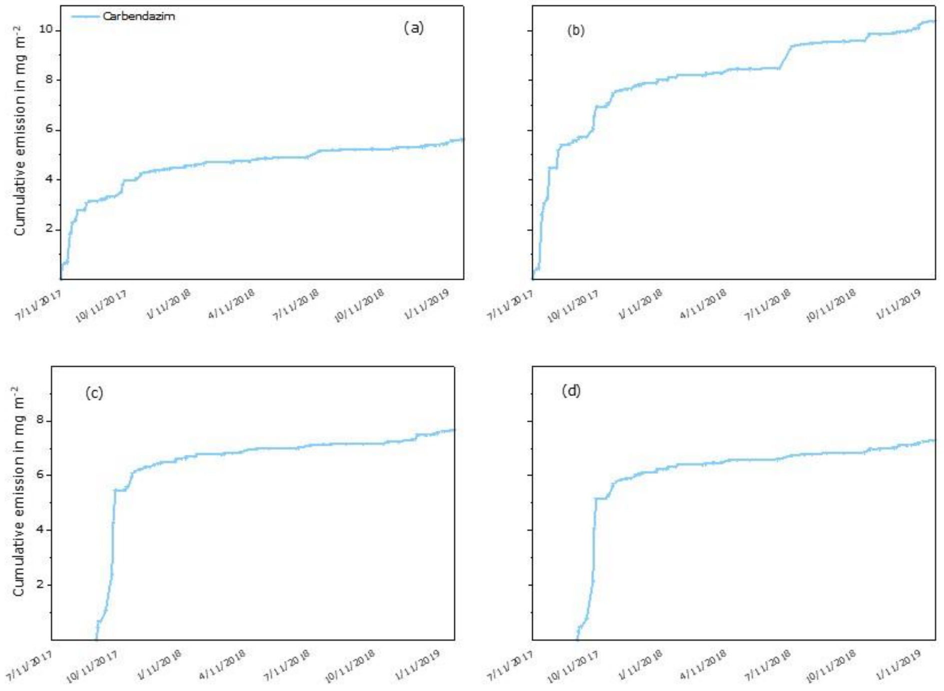

Figure S7. Emission curves of carbendazim, (a) experiment A, white paint, (b) experiment A, red paint, (c) experiment B, white paint, (d) experiment B, red paint.

SI 9: Mass balances

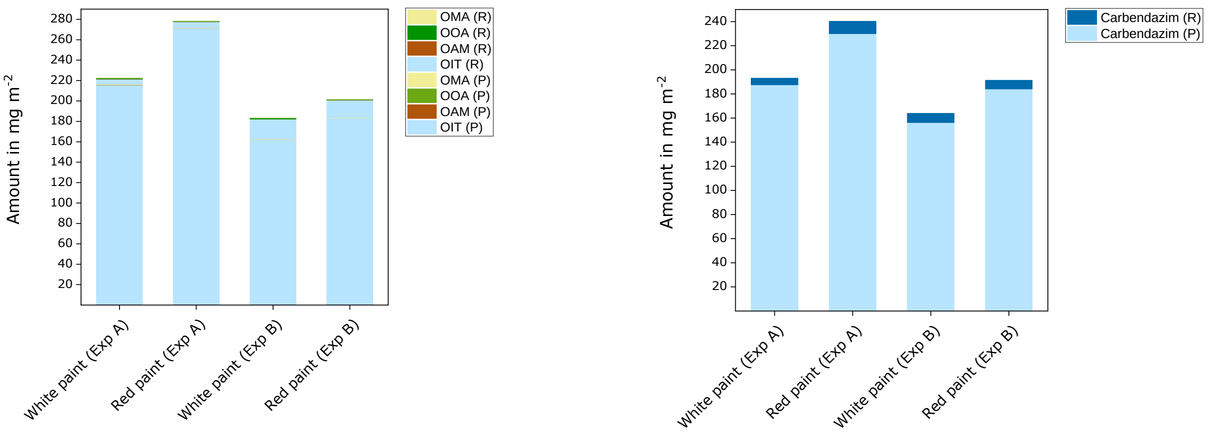

Figure S8. Mass balances of OIT and transformation products (left graph), and carbendazim (right graph) for the white and red test panels at the end of the semi-field experiment. The amounts of the transformation products are related to the initial amounts of the biocidal active substances, i.e., the initial amounts of transformed biocidal active substances were calculated and presented in the graphs. Data for the test panels (P): mean value of six sections, data for the runoff (R): sum of 83 (experiment A) and 69 (experiment B) runoff samples. Calculated original amounts for active substances: 230 mg m^{-2} (experiment A) and 218 mg m^{-2} (experiment B).

References

[1] Schoknecht, U.; Mathies, H.; Lisec, J. Leaching and transformation of film preservatives in paints induced by combined exposure to ultraviolet radiation and water contact under controlled laboratory conditions. *Water* (2021), 13, 2390. doi.org/10.3390/w13172390

[2] Chemspider: http://www.chemspider.com/

[3] Pubchem: https://pubchem.ncbi.nlm.nih.gov/compound/Octylcarbamoyl_formic-acid

[4] Website of the Deutscher Wetterdienst: https://www.dwd.de/DE/leistungen/klimadatendeutschland/vielj_mittelwerte.html and https://www.dwd.de/DE/leistungen/solarenergie/strahlungskarten_mvs.html (assessed on 2022 09 15)

[5] EN ISO 15927-3:2009: Hygrothermal performance of buildings – Calculation and presentation of climatic data – Part 3: Calculation of driving rain index for vertical surfaces from hourly wind and rain data. Beuth Verlag: Berlin, Germany, 2009.

[6] Website of the Umweltbundesamt: https://www.umweltbundesamt.de/sites/default/files/medien/2875/dokumente/umweltqualitaetsnormen_des_chemischen_zustands_0.pdf (assessed on 2022 09 15)

[7] Wünnemann, H., Weiß, K., Arndt, D., Baumann, M., Weiß, R., Ferling, H., Scholz-Göppel, K., Bucher, K., Feick, Ch., Hartmann, G., Kitzing, P., Szyja, M., Schwaiger, J.: Umweltqualitätsnormen für Binnengewässer; UBA-Texte 233/2020; Federal Environment Agency: Dessau-Roßlau, Germany, 2020
